# Prognostic Value of Hemoglobin, Albumin, Lymphocyte, and Platelet Score in Predicting Mortality in Patients With Aortic Dissection: A Retrospective Single‐Center Study Based on ROC Curve Analysis

**DOI:** 10.1155/emmi/6996031

**Published:** 2026-02-24

**Authors:** Canan Şahin, Yahya Şahin

**Affiliations:** ^1^ Department of Emergency Medicine, Emergency Medicine Specialist, Ahi Evran University Faculty of Medicine, Kırşehir, Türkiye, ahievran.edu.tr

**Keywords:** aortic dissection, composite biomarkers, HALP score, hematologic indices, inflammation, mortality prediction, risk stratification, ROC analysis

## Abstract

**Background:**

Aortic dissection (AD) is a life‐threatening cardiovascular emergency associated with high mortality. Early risk stratification through reliable biomarkers is critical for guiding clinical decisions. The hemoglobin, albumin, lymphocyte, and platelet (HALP) score is a novel composite index reflecting inflammation, nutritional status, and hematologic balance. Although it has shown prognostic relevance in several disease states, its utility in predicting mortality in AD remains unknown. This study aimed to investigate the prognostic value of the HALP score and other hematologic markers in patients with AD.

**Methods:**

This retrospective study included 51 patients diagnosed with AD between January 2020 and December 2024 using contrast‐enhanced thoracoabdominal computed tomography. Patients were grouped as survivors or nonsurvivors. Hematologic parameters (hemoglobin, hematocrit, red blood cell, platelet count, and mean platelet volume) and inflammatory indices (neutrophil‐to‐lymphocyte ratio [NLR], platelet‐to‐lymphocyte ratio [PLR], systemic immune–inflammation index [SII], and HALP score) were recorded. The Shapiro–Wilk test assessed normality; Student′s *t*‐test or the Mann–Whitney *U* test was applied accordingly. ROC analysis was performed to evaluate the predictive power of each parameter. Statistical significance was defined as *p* < 0.05. The primary outcome was in‐hospital mortality.

**Results:**

Overall mortality was 33.3%. Nonsurvivors were older and had significantly lower levels of hemoglobin, hematocrit, RBC, and platelet count (*p* < 0.001). The HALP score was lower in the exitus group, though not statistically significant in direct comparison (*p* = 0.549). ROC analysis revealed that the HALP score had an AUC of 0.715 (95% CI: 0.572–0.857, *p* = 0.003), with 55.9% sensitivity and 82.4% specificity at a cutoff of 4.05. Classical parameters such as RBC (AUC = 0.824), Hgb (AUC = 0.802), and Htc (AUC = 0.811) demonstrated stronger predictive capacity. The in‐hospital mortality rate was 33.3%.

**Conclusions:**

The HALP score demonstrated high specificity and moderate sensitivity in predicting mortality in AD, suggesting its potential as a complementary biomarker. Its ease of use and accessibility make it suitable for emergency clinical settings. Prospective multicenter studies are needed to confirm its prognostic validity and routine application in AD management.

## 1. Introduction

Aortic dissection (AD) is a rare but life‐threatening vascular condition with a high mortality rate, characterized by the separation of the aortic wall layers following a tear in the intimal layer. The majority of cases involve male patients over the age of 50, and mortality rates are considerably high [1]. Approximately 49% of acute AD cases die before reaching the hospital, and 30% die during hospitalization [2]. In the Stanford classification, Type A dissection refers to ruptures involving the ascending aorta, whereas Type B dissection refers to those involving the descending aorta. According to the DeBakey classification, Type I dissection involves the ascending aorta, aortic arch, and descending aorta; Type II involves only the ascending aorta; and Type III involves only the descending aorta [1]. Hypertension (HT) is identified as the most prevalent modifiable risk factor, present in approximately 75% of cases. Additionally, atherosclerosis, connective tissue disorders, and prior cardiac surgeries are among other significant risk factors [1]. Although AD is about three times more common in men than in women, it tends to have a poorer prognosis in women [3].

The diagnostic process requires a comprehensive and multidisciplinary approach incorporating clinical examination findings, biochemical markers, and high‐resolution imaging techniques [1]. Although computed tomography (CT) has greatly advanced the evaluation of dissection localization and extent, a substantial reduction in disease‐related mortality has not yet been achieved [4]. This situation has heightened the need for new biomarkers capable of predicting mortality risk and guiding clinicians in early intervention and individualized treatment strategies. Currently, it is well‐supported by numerous experimental and clinical studies that inflammation plays a central and indispensable role in the pathogenesis of cardiovascular diseases [4]. Marked alterations in peripheral blood components including white blood cells (WBCs), neutrophils (NEUs), monocytes (MONOs), platelets (PLTs), and lymphocytes (LYMs) in patients with AD reflect the degree of systemic inflammatory response and the biological behavior of the disease, thereby holding critical prognostic value [5]. However, the limited prognostic utility of isolated biomarkers necessitates the adoption of composite indices that integrate multiple parameters and possess enhanced predictive capacity to support clinical decision‐making. In this context, multiparameter biomarkers such as the neutrophil‐to‐lymphocyte ratio (NLR), platelet‐to‐lymphocyte ratio (PLR), systemic immune–inflammation index (SII), and hemoglobin, albumin, lymphocyte, and platelet (HALP) score (hemoglobin × albumin × lymphocyte/platelet) are gaining increasing significance. The HALP score was first proposed by Jiang et al. in 2015 to predict survival in patients with gastric cancer [6]; in subsequent years, it has been studied across various cancer types as well as cardiovascular and systemic diseases [7]. However, the findings of these studies are frequently inconsistent, sustaining the ongoing debate surrounding the prognostic reliability of the HALP score [8–11].

To date, there are no studies in the literature evaluating the prognostic potential of the HALP score in cases of AD. Addressing this important gap in knowledge, the primary aim of the present study was to investigate the predictive value of blood parameters and the HALP score for mortality in patients with AD. As a secondary aim, the study sought to assess the sensitivity and specificity of the HALP score in relation to mortality using receiver operating characteristic (ROC) analysis and to demonstrate the diagnostic and prognostic applicability of this score in emergency department settings.

## 2. Patients and Methods

### 2.1. Data Collection and Participants

**FIGURE 1:**
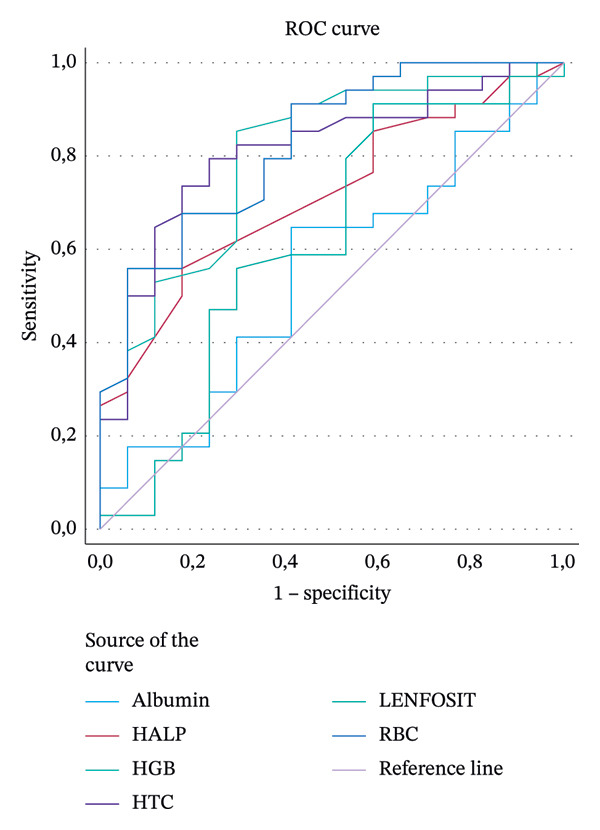
ROC curve.

This study was approved by the Non‐Interventional Clinical Research Ethics Committee of Ahi Evran University Faculty of Medicine (Approval No.: 2025‐10/113). Between January 1, 2020, and December 31, 2024, the records of patients admitted to the Emergency Department of Kırşehir Ahi Evran University Faculty of Medicine Hospital, diagnosed with AD through thoracic and abdominal contrast‐enhanced CT, and subsequently hospitalized in our institution, were retrospectively reviewed. The patient selection process, including inclusion and exclusion criteria, is summarized in the participant flow diagram (Figure 1).

In this retrospective study, all patients who presented to the emergency department and were diagnosed with AD by contrast‐enhanced thoracoabdominal CT constituted the study population. Since the institutional diagnostic protocol confirms AD exclusively through CT, no preliminary screening stage was required, and all CT‐verified cases aged 18 years or older were evaluated for eligibility. The inclusion criteria were as follows: (1) adults aged ≥ 18 years and (2) confirmation of AD via contrast‐enhanced CT in the emergency department. The exclusion criteria were as follows: (1) patients who died before diagnostic imaging could be performed, (2) patients receiving anticoagulant or coagulation‐modifying therapy, and (3) patients transferred to another hospital either for medical reasons or upon personal request. Use of anticoagulant or coagulation‐modifying therapy was predefined as an exclusion criterion. However, because prepresentation medication history was not reliably documented in the electronic hospital system, no patients were excluded based solely on this criterion. All CT‐confirmed cases with complete laboratory data were therefore retained in the final analysis. In addition, patients with clinical conditions known to significantly influence inflammatory markers and hematologic indices—including prior blood transfusion before initial blood sampling, active infection or sepsis, advanced renal or hepatic failure, known malignancy, and chronic use of steroids or other immunosuppressive medications—were also excluded. These factors were omitted a priori to prevent potential confounding effects on the components of the HALP score. Each component of the HALP score was recorded with standardized measurement units: hemoglobin (Hgb) (g/dL), albumin (g/dL), lymphocyte count (× 10^3^/μL), and platelet count (× 10^3^/μL). The HALP score was calculated using the formula HALP = hemoglobin × albumin × lymphocyte/platelet. All measurements were obtained from the initial venous blood samples at emergency department admission, ensuring reproducibility and consistency of the HALP calculation across all patients. Because the hospital information system allowed direct identification of all patients who were admitted to our institution after a confirmed diagnosis, the study universe naturally consisted only of eligible AD cases, and no additional screening or case selection was necessary. There were no patients with missing laboratory or clinical data; therefore, all eligible cases (*n* = 51) were included in the final analysis. In our emergency department, blood samples are routinely drawn immediately upon patient arrival, prior to triage categorization and before any diagnostic or therapeutic intervention. Although the electronic hospital registry records the sample collection time based on the completion of clerical data entry—which may vary depending on patient volume and workflow—the actual blood draw occurs at the moment of arrival as part of the standard emergency protocol. Therefore, all laboratory parameters used in this study reflect the initial physiological state of the patients at presentation. However, the exact time interval between symptom onset and blood sampling could not be reliably obtained from the hospital information system due to inconsistent documentation. In this study, all recorded deaths represent in‐hospital mortality; postdischarge or 30‐day mortality was not included in the analysis.

Baseline clinical characteristics revealed a substantial burden of comorbid conditions among the study population. HT was the most prevalent comorbidity, identified in 28 patients (54.9%). This was followed by hyperlipidemia in 12 patients (23.5%), diabetes mellitus and cardiovascular disease in 10 patients each (19.6%), and cerebrovascular disease in 6 patients (11.8%). These findings indicate that a considerable proportion of patients presented with preexisting systemic diseases, which may have contributed to the clinical course and overall prognosis.

### 2.2. Statistical Analysis

Statistical analyses were performed using IBM SPSS Statistics for Windows, Version 29.0 (IBM Corp., Armonk, NY, USA). The normality of continuous variables was assessed using the Shapiro–Wilk test, while the assumption of homogeneity of variances was evaluated using Levene’s test.

Continuous variables that satisfied the normality assumption were expressed as mean ± standard deviation (SD), whereas nonnormally distributed continuous variables were reported as median (minimum–maximum). Categorical variables were summarized as frequencies and percentages [*n* (%)].

Variables that satisfied both normality and variance homogeneity assumptions, including age, Hgb, hematocrit (Htc), red blood cell (RBC) count, mean platelet volume (MPV), and PLT count, were compared between groups using the independent samples *t*‐test. Variables that did not meet the normality assumption, namely the SII, PLR, and NLR, were analyzed using the Mann–Whitney *U* test.

To evaluate the diagnostic performance of continuous variables for mortality, ROC curve analysis was performed. For variables with statistically significant area under the curve (AUC) values, including HALP, RBC, Hgb, and Htc, the optimal cutoff values, along with corresponding sensitivity and specificity estimates, were determined and summarized. Given the limited number of outcome events, multivariable logistic regression modeling was not performed to avoid overfitting under events‐per‐variable constraints.

All statistical analyses were conducted using a two‐sided significance level of α = 0.05.

## 3. Results

A total of 51 patients who met the specified criteria were included in the study. The mean age of the patients who died was 65.2 years, whereas the mean age of the survivors was 56.3 years. Upon analysis of age distribution, it was observed that the mean age was significantly higher in the deceased group (65.2 ± SD years), suggesting that advanced age may represent a potential risk factor for mortality. In contrast, the mean age of survivors was 56.3 ± SD years, indicating a more favorable prognosis in younger individuals. Of the total patient population, 68.7% were male. Mortality was observed in 33.3% of all cases (Table 1).

**TABLE 1:** Some demographic and clinical data of the patients.

Sex	*n* (%)
Male	35 (68.7)
Female	16 (31.3)
Exitus	17 (33.3)
Survivor	34 (66.7)

*Dissection types*	
Stanford	
Type A	28 (54.9)
Type B	23 (45.1)

DeBakey	
Type I	23 (45.1)
Type II	7 (13.7)
Type III	21 (41.2)

In the study, certain hematologic and demographic parameters were compared between survivors and exitus patients (Table 2). When age was evaluated, the mean age in the exitus group was (SD) 65.23 (14.67) years, indicating that age may have a determining effect on mortality.

**TABLE 2:** Comparison of certain hematological and demographic parameters between the surviving and deceased groups.

Variables	Survivor	Exitus	*p*
Mean age (years)	56.32 ± 11.84	65.23 ± 14.67	0.024^∗^
Hgb (g/dL)	14.25 ± 1.99	12.14 ± 1.62	≤ 0.001^∗^
Htc (%)	42.57 ± 5.39	36.84 ± 3.97	≤ 0.001^∗^
RBC (million/μL)	5.00 ± 0.61	4.19 ± 0.60	≤ 0.001^∗^
MPV (μm^3^)	10.02 ± 0.94	10.65 ± 1.14	0.044^∗^
PLT (thousand/μL)	261.79 ± 95.91	189.17 ± 57.67	0.006^∗^
HALP	0.63 (0.13–5.19)	0.47 (0.09–1.21)	0.549&
SII	705.77 (63.59–3291.67)	637.12 (266.08–2011.95)	0.603&
PLR	98.52 (12.22–305.63)	96.92 (34.78–342.16)	0.549&
NLR	2.84 (0.80–11.95)	3.51 (1.33–14.46)	0.436&

*Note:* Hgb: hemoglobin, Htc: hematocrit.

Abbreviations: MPV = mean platelet volume, RBC = red blood cell.

^∗^Independent *t*‐test, and Mann–Whitney *U* test.

When Hgb levels were assessed, the mean hemoglobin level was significantly higher in survivors (14.25 ± 1.99 g/dL) compared with the exitus group (12.14 ± 1.62 g/dL).

Similarly, the mean hematocrit level was significantly higher in survivors (42.57 ± 5.39%) than in the exitus group (36.84 ± 3.97%).

RBC count is an indirect indicator of tissue oxygenation capacity and peripheral perfusion. The significantly higher RBC level observed in the survivor group (5.00 ± 0.61 million/μL vs. 4.19 ± 0.60 million/μL, *p* ≤ .001) suggests that sufficient oxygen‐carrying capacity may play a decisive role in reducing mortality.

MPV reflects the level of platelet activation and, therefore, the severity of thromboinflammatory processes.

Assessment of PLT is essential in evaluating hemostatic balance and thrombotic risk. The significantly higher PLT count in the survivor group (261.79 ± 95.91 × 10^3^/μL vs. 189.17 ± 57.67 × 10^3^/μL, *p* = .006) suggests that sufficient hemostatic reserve may be protective against mortality.

No statistically significant difference was observed between the groups in terms of the SII (*p* = .603). The mean SII was (SD) 1062.07 (945.70) in the survivor group and (SD) 779.75 (501.60) in the exitus group. Median values were 705.77 (63.59–3291.67) and 637.12 (266.08–2011.95), respectively.

Similarly, PLR values did not show a statistically significant difference between the two groups (*p* = .549). The mean PLR was (SD) 117.80 (68.93) in survivors, with a median of 98.52 (12.22–305.63); in the exitus group, the mean PLR was (SD) 109.29 (74.78), with a median of 96.92 (34.78–342.16).

Regarding NLR, the mean value was 3.87 ± 2.82 in the survivor group and (SD) 4.49 (3.30) in the exitus group; this difference was not statistically significant (*p* = .436). Median NLR values were 2.84 (0.80–11.95) and 3.51 (1.33–14.46), respectively.

The HALP score was (SD) 0.86 (1.06) in the survivor group and (SD) 0.58 (0.33) in the exitus group; however, this difference was not statistically significant (*p* = .549). Median HALP values were 0.63 (0.13–5.19) and 0.47 (0.09–1.21), respectively.

In this study, ROC analysis was applied to objectively assess the diagnostic value of hematologic and composite biomarkers in predicting mortality (Table 3). The findings elucidate in detail the biological determinants of the risk of mortality after dissection.

**TABLE 3:** ROC analysis results.

	AUC	CI 95% lower–upper	Sensitivity	Specificity	Youden index	Cutoff	*p* Value
HALP	0.715	0.572–0.857	0.559	0.824	0.383	4.05	0.003
RBC	0.824	0.705–0.942	0.559	0.941	0.500	4.85	≤ 0.001
Hgb	0.802	0.672–0.932	0.853	0.706	0.559	12.55	≤ 0.001
Htc	0.811	0.688–0.935	0.735	0.824	0.559	39.40	≤ 0.001

*Note:* Hgb: hemoglobin. Htc: hematocrit, Optimal cutoff values were determined using the Youden index (sensitivity + specificity − 1).

Abbreviation: RBC = red blood cell.

**FIGURE 2:**
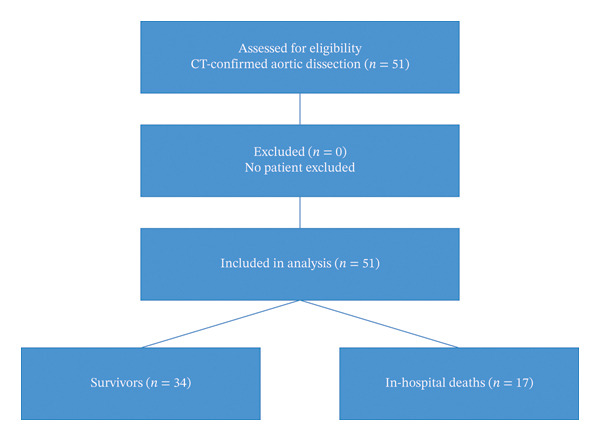
Participant flow diagram of the study cohort.

The HALP score is an innovative index that combines HALP levels, integrating systemic inflammation, nutritional status, and hematologic balance within a single framework. In our study, the AUC of the HALP score was 0.715 (95% CI: 0.572–0.857) and this result was statistically significant (*p* = .003) (Figure 2). The optimal HALP cutoff value of 4.05 was determined using the Youden index (*J* = sensitivity + specificity − 1). At this cutoff value, the sensitivity was 55.9% (95% CI: 38.3%–72.4%) and the specificity was 82.4% (95% CI: 56.6%–96.2%).

In conclusion, our study demonstrated the potential of the HALP score as a meaningful biomarker in predicting mortality in patients with AD, and its high specificity indicated that it is an index that should be considered in clinical decision‐making processes.

## 4. Discussion

Identification of reliable biomarkers for predicting mortality in patients with AD is crucial for early risk stratification and timely therapeutic intervention. Increasing evidence highlights that inflammation and hemostatic imbalance play a central role in the pathogenesis and progression of AD [12, 13]. Consequently, the potential role of inflammatory biomarkers as prognostic indicators in acute AD has attracted growing clinical and scientific interest [14, 15].

The biological plausibility of this relationship is supported by the central role of inflammatory activation, endothelial dysfunction, and impaired tissue perfusion in the progression of acute AD. Elevated inflammatory burden accelerates medial degeneration and increases aortic wall fragility, which may predispose patients to rapid hemodynamic deterioration. Simultaneously, impaired oxygen‐carrying capacity, hypoalbuminemia‐related endothelial leakage, lymphopenia reflecting immune dysregulation, and platelet consumption due to thromboinflammation collectively contribute to worse clinical outcomes. Therefore, biomarkers that integrate these pathways—such as HALP—have a strong theoretical foundation for predicting mortality in AD.

Several studies have examined the prognostic value of composite inflammatory indices such as NLR, PLR, and SII, consistently supporting the impact of inflammation on mortality [16–19]. NLR is widely acknowledged as a robust marker of systemic inflammation, with higher levels correlating with worse outcomes in cardiovascular diseases [20–22]. Zhang et al. reported that NLR levels were significantly elevated in AD patients, and even more so in fatal cases, proposing cutoff values of 5.67 for distinguishing AD from other causes of chest pain and 9.2 for predicting in‐hospital mortality [23]. Bedel et al. and Ustaalioğlu et al. also confirmed elevated NLR as a predictor of mortality [16, 19], while Zhao and Wang observed similar associations [17, 24]. Nevertheless, in our study, NLR did not demonstrate a statistically significant relationship with mortality. This discrepancy may reflect the relatively limited sample size, heterogeneity of patient subgroups, and interpopulation differences in inflammatory responses.

Activated PLTs are recognized not only for their hemostatic role but also as key mediators of inflammation, contributing to pathogenic thrombosis in response to vascular injury [25]. This dual function positions PLR as an integrated marker of thromboinflammatory activity. Xie et al. showed that PLR values outside the 108–188 range were associated with significantly higher mortality [18]. Xie et al. reported elevated PLR in AD patients who developed complications after surgical intervention [17], and Altunova et al. demonstrated that PLR was significantly increased in patients with fatal outcomes [19]. Numerous studies further support the prognostic relevance of platelet‐derived indices in AD [26–28]. However, consistent with our findings for NLR, no significant association between PLR and mortality was identified in our study, most likely due to sample size limitations and the single‐center nature of the research. Larger studies may better clarify its potential prognostic value.

Although AD is a multifactorial disease, the pivotal role of inflammation is now well established [4]. A growing body of literature has demonstrated correlations between inflammatory indices (NLR, PLR, and SII) and adverse outcomes in AD [16–19, 28–30]. Xu et al. found that elevated SIRI and SII were associated with increased mortality and postoperative complications, while Hanna et al.’s meta‐analysis of 16 studies underscored that high SII levels strongly predicted mortality and poor outcomes, and elevated preoperative NLR was linked to postoperative complications and reduced survival [31]. In contrast, our study did not observe a significant relationship between SII and mortality. Possible explanations include sample size limitations, heterogeneity in patient characteristics, and variability in the inflammatory response. Furthermore, differences in cutoff values for these indices across populations suggest limited generalizability.

In contrast to these indices, the HALP score remains relatively underexplored in AD [18]. By integrating HALP values, HALP provides a holistic reflection of inflammation, nutritional status, and hematologic balance. Our study is among the first to evaluate its prognostic potential in AD using ROC analysis. Each component of the HALP score has an established pathophysiological relationship with adverse outcomes in acute AD. Lower Hgb levels reduce oxygen delivery to vital organs, exacerbating tissue hypoxia during hemodynamic instability. Hypoalbuminemia reflects both malnutrition and systemic inflammation and is associated with impaired vascular integrity, increased endothelial leakage, and higher susceptibility to circulatory collapse. Lymphopenia is a marker of immune dysfunction and severe physiological stress, conditions frequently encountered during the acute phase of dissection. Platelet depletion, on the other hand, may indicate consumption due to thromboinflammatory activation, contributing to organ malperfusion and adverse prognosis. By combining these interrelated pathways, the HALP score provides a more integrated assessment of disease severity than any single parameter alone.

The AUC for HALP was 0.715, with sensitivity of 55.9% and specificity of 82.4%. While classical hematologic parameters such as RBC, Htc, and Hgb showed higher AUC values (> 0.80), HALP offered higher specificity, suggesting that it may serve as a valuable complementary tool, particularly in clinical contexts requiring precise risk stratification such as surgical decision‐making. Its accessibility and cost‐effectiveness further enhance its potential applicability in emergency departments and intensive care units.

One of the main limitations of this study is the relatively small sample size and the limited number of in‐hospital mortality events. According to events‐per‐variable considerations, multivariable modeling would have allowed for only a very limited number of covariates and carried a substantial risk of overfitting. Therefore, multivariable regression analyses were not pursued, and the results should be interpreted primarily in terms of univariable associations and ROC‐based discrimination performance. In addition, although a post hoc power analysis for the primary ROC hypothesis (AUC > 0.50) indicated an achieved statistical power of approximately 75%–80%, the limited sample size may still affect the precision of effect estimates.

Recent cardiovascular studies also provide compelling support for HALP as a multidimensional prognostic indicator [32–35]. Liu et al. showed that low HALP scores were associated with increased mortality in STEMI patients, identifying a cutoff of 40.11 [36]. Kiliç et al. demonstrated its prognostic value for early mortality following coronary intervention [37], while Kılıç et al. reported strong associations with short‐ and long‐term mortality in NSTEMI [38]. Similarly, Karakayali et al. demonstrated that the HALP score was an independent predictor of in‐hospital mortality in patients with STEMI undergoing primary PCI, further supporting its role as a multidimensional prognostic marker in acute cardiovascular emergencies [7]. In addition, HALP has been identified as an independent predictor of long‐term mortality in EVAR patients [20]. Since low levels of Hgb, albumin, LYMs, and PLT are each independently linked to adverse outcomes in AD [19, 24, 26, 28, 39], combining these markers into a single index provides a more comprehensive assessment of prognosis.

In our cohort, HALP values were lower in fatal cases, and ROC analysis demonstrated a moderate discriminative ability for mortality prediction, with an AUC of 0.715 (95% CI: 0.572–0.857, *p* = .003), a sensitivity of 55.9%, and a specificity of 82.4% at an optimal cutoff value of 4.05. Although this performance was slightly inferior to classical hematologic parameters such as RBC, Hgb, and Htc in terms of AUC, the relatively high specificity of the HALP score suggests that it may be particularly useful for identifying high‐risk patients who require closer monitoring, early multidisciplinary evaluation, and more aggressive therapeutic strategies. The simplicity and rapidity of its calculation from routinely available laboratory parameters further support its potential applicability as a complementary prognostic tool in emergency department settings. From a clinical standpoint, the HALP score may offer practical value in the early triage of patients with acute AD, particularly in emergency department settings where rapid decision‐making is essential. A low HALP score could help identify patients who are at increased risk of early hemodynamic decompensation and may therefore benefit from immediate multidisciplinary evaluation, more intensive monitoring, or expedited transfer to centers with advanced surgical capabilities. Conversely, patients with higher HALP scores may represent a comparatively lower‐risk group, potentially assisting clinicians in prioritizing resources during acute presentations. Given its simplicity, low cost, and reliance on routinely obtained laboratory parameters, the HALP score may serve as a useful adjunct to established risk assessment tools.

In conclusion, this study highlights the HALP score as an innovative, practical, and valuable biomarker for predicting mortality in AD. While classical indices such as NLR, PLR, and SII showed limited prognostic performance, HALP demonstrated greater specificity, offering a promising supplementary tool for clinical decision‐making. These results contribute original evidence to the literature and provide a strong foundation for future research on integrated prognostic scoring systems in AD.

## 5. Conclusions

This study demonstrates that the HALP score may serve as a valuable and practical biomarker for predicting mortality in patients with AD.

## Funding

This research did not receive any specific grant from funding agencies in the public, commercial, or not‐for‐profit sectors.

## Ethics Statement

This study was approved by the Non‐Interventional Clinical Research Ethics Committee of Ahi Evran University Faculty of Medicine (Approval No.: 2025‐10/113). The ethical approval document is provided as Supporting File 1).

## Conflicts of Interest

The authors declare no conflicts of interest.

## Supporting Information

Additional supporting information can be found online in the Supporting Information section. (*Supporting Information*)

## Supporting information


**Supporting Information 1** Supporting File 1: Ethics committee approval document for the retrospective observational study.

**Supporting Information 2** Supporting File 2: STROBE checklist for reporting of the retrospective observational study.

## Data Availability

The data that support the findings of this study are available from the corresponding author upon reasonable request.
