# Supplementary material for: Prognostic Value of Hemoglobin, Albumin, Lymphocyte, and Platelet Score in Predicting Mortality in Patients With Aortic Dissection: A Retrospective Single‐Center Study Based on ROC Curve Analysis
Source: Emerg Med Int. 2026 Feb 24;2026:6996031. doi: 10.1155/emmi/6996031 (PMC12930098; doi:10.1155/emmi/6996031)
Supplement: Supplementary file 1 — Supporting Information 1 Supporting File 1: Ethics committee approval document for the retrospective observational study. [file EMMI-2026-6996031-s002.pdf]

SAĞLIK BİLİMLERİ BİLİMSEL ARAŞTIRMALAR ETİK KURULU

ARAŞTIRMANIN AÇIK ADI	"Aort Diseksiyonlu Hastalarda Mortaliteyi Öngörmede HALP Skorunun Prognostik Değeri: ROC Tabanlı Tanısal Performans Değerlendirmesi"
VARSA ARAŞTIRMANIN PROTOKOL KODU	

ETİK KURUL BİLGİLERİ	ETİK KURULUN ADI	Kırşehir Ahi Evran Üniversitesi Tıp Fakültesi Sağlık Bilimleri Bilimsel Araştırmalar Etik Kurulu
	AÇIK ADRESİ	Kırşehir Ahi Evran Üniversitesi Tıp Fakültesi Bağbaşı Yerleşkesi Merkez/KIRŞEHİR
	TELEFON / FAKS / E-POSTA	0386 280 3924 / 0386 280 5007 / tipetikkurul@ahievran.edu.tr

BAŞVURU BİLGİLERİ	KOORDİNATÖR/SORUMLU ARAŞTIRMACI UNVANI/ADI/SOYADI	Dr. Öğr. Üyesi Canan ŞAHİN			
	KOORDİNATÖR/SORUMLU ARAŞTIRMACININ UZMANLIK ALANI	Acil Tıp			
	KOORDİNATÖR/SORUMLU ARAŞTIRMACININ BULUNDUĞU MERKEZ	Kırşehir			
	VARSA İDARİ SORUMLU UNVANI/ADI/SOYADI				
	DESTEKLEYİCİ				
	PROJE YÜRÜTÜCÜSÜ UNVANI/ADI/SOYADI (TÜBİTAK vb. gibi kaynaklardan destek alanlar için)				
	ARAŞTIRMAYA KATILAN MERKEZLER	TEK MERKEZ ☒	ÇOK MERKEZLİ ☐	ULUSAL ☒	ULUSLARARASI ☐

BELGELER	Belge Adı	Tarihi	Versiyon Numarası	Dili		
	ARAŞTIRMA PROTOKOLÜ	20.05.2025	1	Türkçe ☒	İngilizce ☐	Diğer ☐

KARAR BİLGİLERİ	**Karar No: 2025-10/113**	**Tarih: 27/05/2025**
	Yukarıda bilgileri verilen başvuru dosyası ile ilgili belgeler araştırmanın/çalışmanın gerekçe, amaç, yaklaşım ve yöntemleri dikkate alınarak incelenmiş ve uygun bulunmuş olup araştırmanın/çalışmanın başvuru dosyasında belirtilen merkezlerde gerçekleştirilmesinde etik ve bilimsel sakınca bulunmadığına, toplantıya katılan Etik Kurul üye tamsayısının salt çoğunluğu ile karar verilmiştir. Ancak Kırşehir İl Sağlık Müdürlüğünden çalışmanın onay yazısı alındıktan sonra çalışmaya başlanabilir.	

Etik Kurul Başkanının
Unvanı/Adı/Soyadı: Doç. Dr. Recai DAĞLI
İmza:

Not: Etik kurul başkanı, imzasının yer almadığı her sayfaya imza atmalıdır.
