# Supplementary material for: Prognostic Value of Hemoglobin, Albumin, Lymphocyte, and Platelet Score in Predicting Mortality in Patients With Aortic Dissection: A Retrospective Single‐Center Study Based on ROC Curve Analysis
Source: Emerg Med Int. 2026 Feb 24;2026:6996031. doi: 10.1155/emmi/6996031 (PMC12930098; doi:10.1155/emmi/6996031)
Supplement: Supplementary file 2 — Supporting Information 2 Supporting File 2: STROBE checklist for reporting of the retrospective observational study. [file EMMI-2026-6996031-s001.docx]

STROBE Checklist
Prognostic Value of Hemoglobin, Albumin, Lymphocyte, Platelet Score in Aortic Dissection

1. Title and abstract – Reported (Title, Abstract)
2. Background/rationale – Introduction
3. Objectives – Introduction (last paragraph)
4. Study design – Patients and Methods
5. Setting – Patients and Methods
6. Participants – Inclusion/Exclusion criteria
7. Variables – HALP, NLR, PLR, SII definitions
8. Data sources – Laboratory and CT
9. Bias – Exclusion of infection, cancer, immunosuppression
10. Study size – All eligible CT-confirmed cases (n=51)
11. Quantitative variables – Statistical Analysis
12. Statistical methods – Statistical Analysis
13. Participant flow – Figure 2
14. Descriptive data – Table 1
15. Outcome data – Results
16. Main results – Tables 2 and 3
17. Other analyses – ROC analyses
18. Key results – Discussion
19. Limitations – Discussion
20. Interpretation – Discussion
21. Generalisability – Discussion
22. Funding – Funding Statement
